# The Expression of Embryonic Liver Development Genes in Hepatitis C Induced Cirrhosis and Hepatocellular Carcinoma

**DOI:** 10.3390/cancers4030945

**Published:** 2012-09-18

**Authors:** Martha Behnke, Mark Reimers, Robert Fisher

**Affiliations:** 1 Transplant Program Administration, Virginia Commonwealth University Health System, 1200 E. Broad St., Richmond, VA 23298, USA; 2 Virginia Institute for Psychiatric and Behavioral Genetics, Virginia Commonwealth University School of Medicine, 800 E Leigh St., Richmond, VA 23298, USA; E-Mail: mreimers@vcu.edu; 3 Department of Surgery, Virginia Commonwealth University, 1200 E. Broad St., Richmond, VA 23298, USA; E-Mail: rafisher@vcu.edu

**Keywords:** hepatocellular carcinoma, hepatitis C, microarray analysis

## Abstract

Hepatocellular carcinoma (HCC) remains a difficult disease to study even after a decade of genomic analysis. Patient and disease heterogeneity, differences in statistical methods and multiple testing issues have resulted in a fragmented understanding of the molecular basis of tumor biology. Some researchers have suggested that HCC appears to share pathways with embryonic development. Therefore we generated targeted hypotheses regarding changes in developmental genes specific to the liver in HCV-cirrhosis and HCV-HCC. We obtained microarray studies from 30 patients with HCV-cirrhosis and 49 patients with HCV-HCC and compared to 12 normal livers. Genes specific to non-liver development have known associations with other cancer types but none were expressed in either adult liver or tumor tissue, while 98 of 179 (55%) genes specific to liver development had differential expression between normal and cirrhotic or HCC samples. We found genes from each developmental stage dysregulated in tumors compared to normal and cirrhotic samples. Although there was no single tumor marker, we identified a set of genes (Bone Morphogenetic Protein inhibitors *GPC3*, *GREM1*, *FSTL3*, and *FST*) in which at least one gene was over-expressed in 100% of the tumor samples. Only five genes were differentially expressed exclusively in late-stage tumors, indicating that while developmental genes appear to play a profound role in cirrhosis and malignant transformation, they play a limited role in late-stage HCC.

## 1. Introduction

### 1.1. Background and Motivation

Hepatocellular Carcinoma (HCC) ranks fifth among all cancers and third in mortality, accounting for hundreds of thousands of deaths per year. One-year survival rates remain less than 50% in the United States, despite advances in therapy [1]. HCC is the primary cancer morbidity evolving over decades in underlying hepatitis C (HCV) liver pathology in North America and Japan. HCC development is generally thought to be a multistep process resulting from hepatocyte turnover, chronic inflammation, regeneration, oxidative stress, DNA damage, and cirrhosis, as well as direct viral injuries. Unfortunately, the specific molecular mechanisms underlying hepatocarcinogenesis remain unclear.

In the last ten years, microarray technology has been a powerful tool to study the molecular basis of disease. By measuring whole-genome transcript levels, expression patterns associated with liver dysfunction have been examined. However, HCC remains a difficult disease to study, with widely variable findings between studies and several proposed non-overlapping gene signatures [2,3,4,5,6,7,8,9,10,11,12]. This is likely due not only to the biological heterogeneity of HCC pathogenesis, but also reflects the varied clinical background of patients and variation in statistical technique. There are significant statistical challenges which plague the analysis and interpretation of microarray experiments. Differences in technique in every stage of data pre-processing have been demonstrated to dramatically affect the end results, including background correction [13], normalization [14,15], and probe set summarization [16].

In addition, traditional statistical approaches are not particularly well-suited to cancer genomic data. When simultaneously comparing many thousands of genes, multiple testing problems are considerable. Worse, because the assumption of independent tests is often violated, actual false positive rates can be much higher than estimated by standard methods [13,14,15]. This implies that, even using conservative multiple testing correction methods, cancer studies which generate thousands of significantly differentially expressed genes could have several hundred false positive results. To reduce this problem, we used a knowledge-driven approach, using what is already known about normal and disease processes to generate hypotheses that can be tested with a relatively small number of statistical tests [17,18,19].

Another difficulty stems from the heterogeneity of cancer processes, in which changes in the expression of important genes occur only in subsets of tumors. This results in skewed density curves (sometimes even bi-modal) that may not be easily detected by means-based tests. Most statistical tests in common use are based on comparing the magnitude of mean change relative to the variation. These tests also place focus on the largest magnitude changes which are often products of tumor behavior, such as increased metabolism and cell proliferation/turnover, rather than drivers that often have smaller fold-changes [20]. We suspect that there are modest changes in the expression of critical genes that may be difficult to distinguish from “noise” in the data, but may have a significant impact on tumor development [21,22]. To address this we applied techniques that find patterns in the data (such as dimension reduction techniques), focused on such genes, to identify those that drive tumor behavior even if they have modest expression changes or skewed distribution patterns.

### 1.2. Questioning Biological Randomness in HCV-HCC

Tumors are widely believed to arise through an accumulation of random mutations. Mutations and chromosomal instability have been demonstrated in several carcinomas, including non-Hodgkins lymphoma, ovarian, colorectal, and oral tissue types [23,24,25,26,27]. HCC arising from chronic HBV infection has also been associated with mutations via HBx gene interference with *p53* binding, leading to faulty DNA repair mechanisms [28,29]. It would be natural to think that the widespread dysregulation of gene expression in HCV-HCC is also largely random. However, HCV-HCC may be unusual because hepatitis C is an RNA virus that codes proteins that have direct interaction with over thirty host proteins. Tumors emerge from an environment of decades of host response to infection and liver damage. Therefore we hypothesize that induction of HCC in chronic HCV liver pathology may depend more on host response to chronic infection and HCV-host interactions than on direct DNA damage. If this is true, the effects of the HCV virus will be seen in the perturbation of the “tools at hand”: gene expression changes that might be expected include modified expression of genes already in use in the liver (including genes expressed by activated hepatic stellate cells), target genes of host proteins that HCV proteins interact with, and genes used in the liver’s own life history. Such genes contain the specific transcription factor binding sites (TFBS) that are responsive to the transcription factors expressed in the liver, while genes that are not normally expressed in the liver are responsive to different promoters. For instance, the promoter region for FGF7 (expressed in the embryonic liver) contains binding sequences for ATF2, FOXD1, HNF3B, STAT3, and JUN which are all expressed in the liver and dysregulated in liver disease. This reasoning also implies that genes never expressed by a healthy liver would not be expected to be activated by HCV-induced tumors to the same extent as in HBV-HCC or other cancers.

To further target our hypotheses, we reviewed the current knowledge of processes involved in HCC. For instance, it has recently been noted that there appear to be pathways common to both cancer and embryonic development in HCC and other cancers [30,31]. In the context of the hypothesis of non-random response to HCV as described above, this led us to question whether any developmental genes involved in HCC are specific to liver development, and if paralog genes (similar in structure and function in other tissues) remain dormant. In this paper we demonstrate that HCV-induced liver cirrhosis and HCC do indeed show a general pattern of differential expression of liver development genes compared to paralog genes that have similar roles in the development of other tissues. Many of these developmental genes are up- or down-regulated in cirrhotic livers in a coherent way (clustering closely together), then degenerating into widely variable expression patterns in tumors. Some of the genes identified in this manner are already associated with HCC, while others appear to be novel. We also observed that some of these important embryonic signals are secreted from mesodermal tissues during development. These same signaling molecules may be secreted from mesodermally-derived stellate cells in adults. However, these cells comprise less than five percent of adult liver volume, which may result in an observed low signal that may have been difficult to distinguish from noise in previous studies.

### 1.3. Overview of Liver Development

Liver development is a multi-stage process orchestrated by nearly 200 master regulators, growth factors, and their receptors. Growth factors secreted externally and from within the developing liver bind receptors on the surface of liver cells, which transduce signals to transcription factors (TFs) within the nucleus. These transcription factors, either individually or as co-factors, regulate a complex program of inducing or repressing access to gene transcription by a number of activities including chromatin decompaction, recruitment of chromatin remodeling complexes, and histone marker methylation, demethylation, or acetylation, as well as by physically blocking or recruiting RNA polymerase. For example, some of the earliest TFs that induce hepatic fate from the endoderm (*FOXA1-3*, *GATA4/6) *open the chromatin structure around early hepatic markers such as albumin and alpha fetoprotein (*AFP)*, while β-catenin (*CTNNB1)*, *LEF1*, and *TCF3* recruit chromatin remodeling complexes, and *ARID5B *and *ATF2* modify histone markers of target genes. See Si-Tayeb *et al*. for a recent review [32] of embryonic liver development.

In the neonatal period, many of these regulators are repressed in most parts of the liver, while others maintain some level of activity throughout adult life either playing roles in metabolism and homeostasis or in maintaining niches of hepatic stem cells.

## 2. Results

### 2.1. Description of Patient Population

Microarray studies were obtained over a 10 year period from 180 samples of cirrhotic tissue and tumors collected from 140 patients with chronic HCV infection. As described in the Experimental Section, stringent quality control criteria were applied minimize technical artifacts, leaving us with 30 HCV-cirrhosis (CIR) and 49 HCV-HCC (HCC) tumors. These were compared to a control group of 12 samples taken from non-diseased, deceased donor livers (NOR). Tissue was obtained from 31 early HCC (stage T1 and T2) and 18 late HCC (T3 and T4). Twenty-nine HCC patients were transplanted, six died on the waiting list and 14 were never listed for transplant due to age, stage of cancer, or other co-morbidities.

### 2.2. Differentially Expressed Genes Are Specific to Liver Development

We hypothesized that genes not normally expressed in adult livers are less likely to be transcriptionally activated in HCV-HCC. To test this, we identified a set of genes with zero counts in an RNA sequencing study on a normal liver sample. 1,399 were represented on the Affymetrix U133Av2 genechip. We tested for expression in HCC compared to normal samples in our dataset using a one-sided Kolmogorov-Smirnov (K-S) test of identical distribution. Of the 1,399 genes, none were expressed in the HCC samples (α < 0.001).

**Figure 1:**
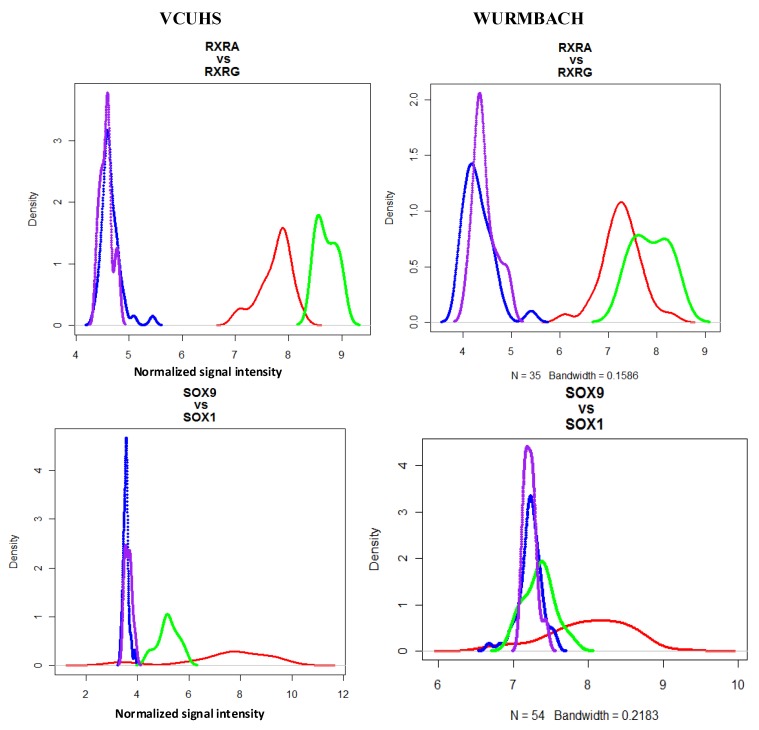
Selected density plots of liver development *vs*. paralog non-liver development genes. Expression densities are shown for gene pairs in normal and HCC samples from our data and in the Wurmbach dataset. Red = expression of the liver gene in HCC; Green = expression of liver gene in normal controls; Blue = expression of non-liver gene in HCC; Purple = expression of non-liver gene in normal controls. Paralog genes (*RXRG* and *SOX1*) were not expressed in HCC or normal samples, while liver development genes *RXRA* and *SOX9* were differentially expressed in HCC. These patterns were also observed in the Wurmbach dataset.

More specifically, we wished to determine whether the general developmental pathways altered in HCC were using genes specific to liver development, or whether any member of the gene family might be engaged. To examine this, we identified 26 paralog genes that have highly related developmental functions in other tissues that are not expressed in normal healthy livers (based on the RNA sequencing data and verified with a literature search). In our data, no paralogs were expressed in HCC compared to normal samples (α < 0.001) (Supplementary Table S1, column A). We validated this to an independently collected HCV-HCC dataset from Wurmbach *et al.* (see Experimental Section), which also had no expression of these paralog genes (Supplementary Table S1, column B). Next, we compared the expression patterns of liver genes to their non-liver paralogs in tumors. Most (31/33, 94%) of the liver genes had significantly different expression distributions compared to their paralog (Supplementary Table S1, column C). Figure 1 shows examples of the typically observed patterns of expression and poor regulation. Even when a liver gene is not differentially expressed in HCC, it often seems to be more poorly regulated with a wider distribution pattern than is seen in normal tissue.

### 2.3. Patterns of Expression in HCV-Cirrhosis and HCV-HCC

We then identified patterns of expression for liver development genes in HCV-CIR and HCV-HCC. To capture changes in either mean or variation, we assessed significance with a combined *p*-value from both *t *and F tests. We were particularly interested in whether the genes associated with progression from cirrhosis to cancer were those particular to a certain developmental stage. However, differentially expressed genes were found from all stages of development, and PCA plots of stage-specific genes all showed discrimination between normal, cirrhotic, and tumor samples (data not shown).

Twenty-nine developmental genes had a pattern of higher magnitude over-expression in cirrhosis, then declining values in HCC (Table 1). These include several extra-cellular matrix (ECM) genes and members of the TGFβ/BMP signaling pathway. Transcription factors following this expression pattern include *SOX9*, *GATA6*, *HAND2*, and *IRS2.* The only genes differentially expressed between early (T1 and T2) and late-stage (T3 and T4) tumors were *EPCAM *and tumor suppressor *KLF6.* Nine genes were down-regulated in cirrhosis and remained low in tumors: transcription factors *FOXA1*, *FOXA2*, *GATA4*, *HNF1A *and *STAT3*; growth factor receptors *ACVR2B*, *RXRA*; signaling molecule *NRTN*, and *MMP15*, which degrades intracellular matrix proteins.

**Table 1:** Liver development genes with significantly higher expression in cirrhosis than tumor samples. Fold-changes are relative to normal samples.

Gene	Gene Name	Gene Function	Mean FC	Mean FC	Mean FC
			CIR	Early HCC	Late HCC
EPCAM	Epithelial cell adhesion molecule	ECM	14.8	14.0	5.7 *
MMP7	Matrix metalloproteinase 7	ECM	7.5	6.1 *	3.5 *
KRT19	Cytokeratin-19	Epidermal IF	6.0	2.9 *	1.8 *
MMP2	Matrix metalloproteinase 2	ECM	5.4	4.8 *	3.0 *
VIM	Vimentin	Mesenchymal IF	5.8	5.2 *	4.8 *
SOX9	SRY-box 9	TF	4.9	3.2 *	2.9 *
LAMA2	Laminin alpha 2	ECM	4.4	2.3 *	1.9*
FGFR2	Fibroblast Growth Factor Receptor 2	GF receptor	4.3	2.4 *	1.8 *
KLF6	Kruppel-like factor 6	TF	3.9	2.6 *	1.8 *
COL4A2	Collagen IV alpha 2	ECM	3.9	2.6 *	2.3 *
LAMB1	Laminin beta 2	ECM	3.5	2.8 *	1.5 *
ARID5B	AT rich interactive domain 5B	TF	3.4	1.8 *	1.7 *
FSTL3	Follistatin-like protein 3	GF antagonist	3.4	1.7 *	1.5 *
TGFB1	Transforming growth factor, beta 1	GF	3.2	2.1 *	1.5 *
SMAD7	SMAD family member 7	Signal transduction	3.2	1.9 *	1.4 *
CITED2	CBP/p300-interacting transactivator	TF	2.8	2.0 *	1.7 *
GATA6	GATA binding protein 6	TF	2.8	1.6 *	0.9 *
SFRP5	Secreted frizzled-related protein 5	Wnt inhibitor	2.6	1.8 *	1.5 *
ID3	Inhibitor of DNA binding 3	TF antagonist	2.4	1.7 *	1.3 *
LAMC3	Laminin gamma 3	ECM	2.4	1.9 *	1.3 *
HAND2	Heart- and neural crest derivatives-expressed protein 2	ECM	2.2	1.7 *	1.4 *
NDN	Necdin	TF	2.2	1.3 *	1.3 *
PTN	Pleiotrophin	GF	2.1	1.6 *	1.4 *
ZBTB20	Zinc finger and BTB domain containing 20	TF	2.1	1.4 *	1.3 *
CDH1	Cadherin 1	ECM	1.8	1.5 *	1.5 *
FGF7	Fibroblast growth factor 7	GF	1.7	1.3 *	1.2 *
BMP2	Bone morphogenic protein 2	GF	1.6	1.4 *	1.3 *
COL4A4	Collagen IV alpha 4	ECM	1.6	1.2 *	1.2 *
CSNK1D	Casein kinase I isoform delta	kinase	1.5	1.1 *	1.0 *
IRS2	Insulin receptor substrate 2	GF receptor	1.4	1.1 *	1.1 *

* denote genes that are differentially expressed compared to cirrhosis (α < 0.001). Abbreviations: TF = transcription factor; ECM = Extra-cellular matrix; IF = intermediate filament; GF = growth factor.

Sixteen genes were differentially expressed uniquely in the tumors (Table 2). Magnitude of change was modest, however six of the 16 genes were transcription factors or activators (*TBX3*, *HHEX*, *ATF2*, *FOXM1*, *PROX1* and *STAT3)* which might be expected to induce large effects with small expression changes. Thirty-five more genes were differentially expressed in cirrhosis and either had similar expression in tumors (25 genes) or had larger magnitude changes in HCC (10 genes). For example, GPC3 was up-regulated slightly in cirrhosis (×2), and greatly up-regulated in both early (×7.2) and late (×10.4) tumors.

**Table 2:** Genes uniquely changed in HCC (α < 0.001).

Gene	Gene Name	Gene Function	FC Early HCC	FC Late HCC
DKK1	Dickkopf-related protein 1	Wnt inhibitor	3.8	1.8
MMP1	Matrix metalloproteinase 1	ECM	2.4	1.5
FST	Follistatin	GF antagonist	1.9	2.1
TBX3	T-box 3	TF	1.3	2.2
MAP4K4	Mitogen-activated protein kinase kinase kinase kinase 4	kinase	1.3	1.3
INHBA	Activin	GF	1.3	1.3
HHEX	Hematopoietically expressed homeobox	TF	1.3	1.3
ATF2	Activating transcription factor 2	TF	1.3	1.4
BSG	Basigen	ECM receptor	1.3	1.4
LAMA4	Laminin alpha 4	ECM	1.2	1.3
FOXM1	Forkhead box M1	TF	1.2	1.1
KRAS	v-Ki-ras2 Kirsten rat sarcoma viral oncogene homolog	GTPase	0.9	0.7
PROX1	Prospero homeobox 1	TF	0.7	0.5
TGFBR3	Transforming Growth Factor beta receptor 3	GF	0.7	0.6
MST1	Macriogage stimulating 1 (hepatocyte growth factor-like)	GF	0.6	0.6
STAT3	Signal transducer and activator of transcription 3	TF	0.6	0.5

Abbreviations: TF = transcription factor; ECM = Extra-cellular matrix; GF = growth factor.

Overall, 98 of the 179 (55%) genes critical to liver development had altered expression patterns in cirrhosis and early stage tumors. Only five of the genes were uniquely changed in late-stage tumors: *ACVR1*, *HMGA2*, *IGF2*, *CP* and *YAP1*. A complete list of all 179 liver development genes tested, and relative expression in each disease group can be found in Supplementary Table S2.

### 2.4. Functional Gene Sets That Discriminate between Normal, Cirrhosis, and Tumor Samples

**Figure 2:**
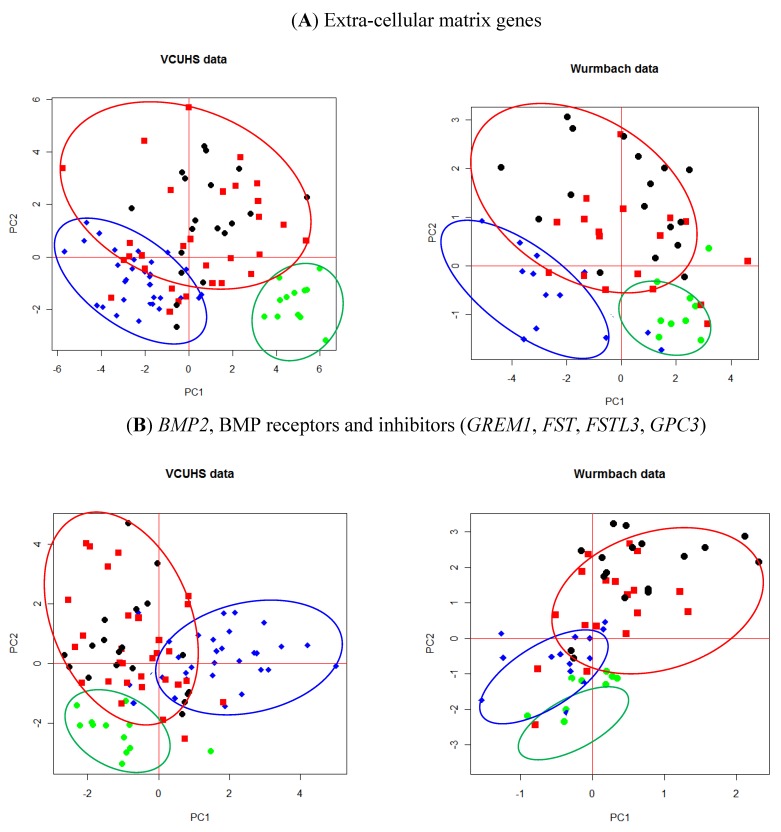
PCA plots of (**A**) ECM genes and (**B**) *BMP2 *and its receptors and inhibitors. Green = normal control livers; Blue = cirrhosis; Red = early stage HCC; Black = late stage HCC. Normal tissues cluster well away from either cirrhosis or tumors. Both the ECM genes (**A**) and BMP inhibitors (**B**) were able to discriminate between cirrhosis and many of the tumor tissues.

From these significant liver development genes we have been able to identify some specific functional groups. Genes related to extra-cellular matrix (ECM) maintenance or remodeling demonstrated major changes in both cirrhosis and tumors. PCA of the significantly changed genes demonstrate that these genes also independently discriminate between normal, cirrhosis, and tumor samples (Figure 2A). The BMP signaling pathway is also highly dysregulated in HCV-cirrhosis and HCC. *BMP2*, its receptors, and BMP inhibitors are all differentially expressed in cirrhosis and HCC. Embryonically, BMP signaling is antagonistic to FGF signaling and this balance is controlled by the DAN family of BMP antagonists from mesenchymal cells and *GPC3 *expressed by hepatocytes. *BMP2*, *BMPR1A*, *FGF7*, *FGFR2* and *ID3* were more highly expressed in cirrhosis than HCC, while the BMP inhibitors were more highly expressed in tumors. At least one of the inhibitors *GPC3*, *GREM1*, *FSTL3*, and/or *FST* were over-expressed (FC > 1.5) in 100% of tumor samples (Supplementary Table S3). A PCA plot demonstrates the gene set’s ability to discriminate most tumors from normal and cirrhosis samples (Figure 2B). Leave-one-out cross-validation (LOOCV) of ROC curves assessed predication sensitivity of 95.9% and specificity of 83.3%.

These findings were validated against the Wurmbach dataset by applying the PCA loadings generated from our data to their dataset, and we observed similar patterns of separation between normal, cirrhosis, and HCC tissues (Figure 2A,B). The ECM and BMP genesets were well described with the first two principal components.

## 3. Discussion

Hepatocellular carcinoma is widely recognized as a highly heterogenous disease, which has made it difficult to characterize. However, because HCV is RNA virus that exerts its effects by direct protein interactions and host response to chronic infection and inflammation, we questioned the common perception that the gene expression changes observed in tumors are likely due to random activation of genes arising from mutations and DNA damage. We screened a total of 1,425 genes that were demonstrated by RNA seq analysis to have no expression in healthy liver. None of them were expressed in our HCV-HCC samples. This data suggests that the progression from cirrhosis to HCC in patients with chronic HCV infection is not accompanied by random gene activation, as has often been supposed. In particular, 26 of these genes have developmental roles highly paralogous to liver development genes and known association with carcinoma of other tissues, but remain under tight transcriptional control in both cirrhotic and HCC tissues. For example, *BMP3* inactivation is associated with colorectal cancer [33], gastric cancer [34], and pancreatic cancer [35]; *CDH3 *is over-expressed in several types of cancer including breast [36] and pancreatic [37]; *FGF3* is amplified in ovarian [38], breast [39], and bladder cancer [40]; *FGF12* is amplified in esophageal squamous cell carcinoma [41] and squamous cell carcinoma of the lung [42], and *GATA1* mutations have recently been associated with acute megakaryoblastic leukaemia [43] and breast cancer [44].

Using a knowledge-driven approach, we have shown that genes critical to every stage of liver development are differentially expressed in HCV-cirrhosis and further deregulated in tumors. In general we observed a pattern of modest changes in genes differentially expressed in cirrhosis and HCC. Mean fold-change in tumors was <2× up or down in many cases. Some of these genes are expressed by mesodermally-derived tissues in the embryonic liver and thus may also be expressed exclusively by the activated hepatic stellate cells (HSCs) in the adult liver. Since these cell types make up less than five percent of the liver volume in HCC, even large changes in gene expression from the HSC would result in modest overall signal change in a tissue sample. *BMP2*, *BMP4*, *BMPR1A*, *HGF*, *LHX2*, *FST*, *SOX9*, *GREM1*, *FGF7*, *TGFB1*, *MMP2*, *TIMP2*, *SMAD7*, *KLF6* and *JUN* have experimentally validated expression in activated hepatic stellate cells [45,46,47,48,49]. All of these genes except *BMP4*, *HGF*, and *LHX2* were differentially expressed in our study. As predicted, most of these have modest but significant mean fold-change.

The major pattern that emerged from our detailed analysis of these important genes was the BMP pathway. Embryonically, *BMP2* and *BMP4 *are secreted from the mesenchymal STM and act antagonistically against *FGF2* and *FGF4 *to regulate rate of proliferation. Later in development, *FGF7* and *FGFR2 *promote differentiation into biliary epithelial cells. In healthy adults, *BMP2* is secreted from hepatic stellate cells to suppress hepatocyte proliferation. During normal liver regeneration (such as after partial hepatectomy), *BMP2* is down-regulated and hepatocyte proliferation is activated by at least four distinct and redundant mechanisms: *FGF7* and *FGFR2* [50]; *KDR* (aka VEGFR2), *ID1*, *HGF*, and *WNT2* [51]; *TNF*, *NFKB1*, *STAT3*, and *IL6* [52]; and β-catenin and Cyclin D1 [53]. During active hepatitis C infection, the HCV core protein induces over-expression of *BMP2*, which then participates in the activation of hepatic stellate cells and also functions to suppress hepatocyte proliferation in response to liver damage. In our dataset, late-stage cirrhotic livers had elevated levels of *BMP2*, its receptor, and some of its downstream effectors (SMADs and ID transcription factors). The proliferation promoters noted above were generally unchanged in cirrhotic tissue, while TNF and β-catenin were under-expressed. *FGF7* and *FGFR2*, however, were over-expressed in both cirrhosis and HCC. This suggests a complex and somewhat chaotic response to active HCV infection and liver damage in the cirrhotic liver. However, many cirrhotic tissues and every tumor in our samples over-expressed at least one of the BMP inhibitors (FC > 1.5, see Supplementary Table S3), with higher expression in tumors. This data suggests there is a chronic struggle in the cirrhotic liver to maintain homeostasis of the BMP pathway, and that tumors might have found a way to overcome the proliferative inhibition imposed by *BMP2 *by sufficiently up-regulating any of the BMP inhibitors. In the tumors, most of the proliferation promoters had expression values similar to that of normal tissue (which is sufficient to induce proliferation in the absence of active *BMP2*), including those that were down-regulated in cirrhosis.

Several of the developmental genes expressed in adult livers have been shown to have roles in maintaining tissue differentiation or regulating cell proliferation. BMPs and their DAN family antagonists have opposing effects on Wnt signaling and the *BMP-GREM-Wnt* circuit has been proposed as a mechanism to maintain stem cell niches in the colon [54]. *FOXA1*, *FOXA2*, *HLX* and *SFRP5* are expressed in progenitor cells, while *SOX9*, *SOX17*, *FOXA2* and *KIT *have confirmed expression in hepatic stem cells [55]. *FOXA1-3* drive differentiation of hepatocyte stem cells. Merlin (*NF2*) has recently been proposed as a regulator of liver stem cells, with deletion leading to HCC in rat models [56]. In our data, *FOXA1*, *FOXA2*, *MST1 *and *NF2* were down-regulated, suggesting that in this population there is a general pattern of de-differentiation of hepatocytes within tumors and acquisition of stem-cell-like properties.

## 4. Experimental Section

### 4.1. Study Population

Since 1997, HCV patients diagnosed with cirrhosis and HCC have been evaluated and treated at the Hume-Lee Transplant Center at VCUHS according to an Institutional Review Board approved study protocol [57]. Informed consent was obtained from all patients. After staging, HCC patients had their tumors ablated and were evaluated for liver transplant according to the United Network for Organ Sharing criteria. Tissue samples were collected from biopsies and explanted livers according to protocols established by the Liver Tissue Cell Distribution System (Richmond, Virginia, funded by NIH Contract #N01-DK-7-0004/HHSN267200700004C). Control liver samples were obtained from explanted donor livers. Donor livers were shown to have normal function and were negative for hepatitis C virus antibodies.

An independently published dataset of HCV-cirrhosis and HCC was also obtained for verification of results [58] (Wurbach *et al.*, NCBI GEO database accession GEO6764). In addition, the absolute expression levels of target genes in a normal adult human were obtained from the BodyMap gene expression database [59] and mapped with Burrows-Wheeler Alignment tool (BWA) [60] with default parameters. In cases where BodyMap results were inconclusive (counts of 0–40), a literature search was performed to confirm adult expression of target genes.

### 4.2. Sample Preparation

Pre-transplant biopsies and explanted livers were sectioned and grossly examined. Samples from tumors and cirrhotic liver tissue (according to diagnosis and pathological examination) were freshly snap-frozen and processed in the Hume-Lee Transplant Center Molecular Diagnostic Laboratory. Liver tissue samples were collected in liquid nitrogen or RNAlater solution (Ambion, Austin, TX, USA) and stored at −80 °C until use. Explanted livers were sliced at intervals of 4–5 mm, and all nodules suspicious for HCC processed for light microscopy. Only tumor samples with more than 85% tumor cell content were used for the microarray studies. Normal and necrotic tissues were macro-dissected from the sample.

With minor modifications, the sample preparation protocol follows the Affymetrix GeneChip Expression Analysis Manual (Affymetrix, Santa Clara, CA, USA). After hybridization and scanning, the microarray images were checked for major chip defects or abnormalities in the hybridization signal. Total RNA quality and integrity of each sample were analyzed using the Agilent 2100 Bioanalyzer (Agilent Technologies), and products of cDNA synthesis and *in vitro* transcription (IVT) were tested before being considered for microarray analysis using the Agilent 2100 Bioanalyzer (cDNA synthesis 1.5 kb < cDNA < 5.0kb; IVT 1.0kb < cRNA < 4.5 kb).

All chips in the study were examined with several quality control tests [61,62]. Any chip that fell well outside the recommendations for any of the quality assessment tests was excluded from further analysis.

### 4.3. Statistical Methods

Data files were read into the R (version 2.13) programming environment using updated probe annotations from the BrainArray project (version 14.1.0, HGU133A2_Hs_REFSEQ), which have been shown to improve accuracy of probe—gene mapping over the standard Affymetrix annotation [63].

Robust Multichip Average (RMA) pre-processing is broadly accepted as robust, easy to implement, and widely applicable. However, we were concerned that the assumptions of RMA may not be met (that only a small proportion (1–5%) of genes are differentially expressed, and that about the same number of genes are over- *vs*. under-expressed). Therefore, we processed a test dataset using RMA and carefully examined differential expression results. Comparison of group contrasts identified 25–45% genes that were called as differentially expressed at an FDR < 0.05, and 2/3 of those genes were over-expressed in tumors. This suggested to us that quantile normalization may not be an appropriate method for this dataset. Instead, we first performed background correction, then normalized the data using non-parametric, distribution-free regression on technical covariates of probes (GC content, melting temperature, and probe location) to estimate and correct for systematic bias [64].

We used scaled Principal Components Analysis (PCA) on specific gene sets to explore the relationship between those genes and disease behavior. We used the function “prcomp” with scale = TRUE in the R environment [65].

Normally regulated genes generally have a narrow distribution with small variation, while poorly regulated genes may have broad, flat distribution of expression values or long thick tails and high variability. Genes that are differentially expressed in a subset of tumor samples will have a skewed distribution. Either of these situations violate the assumption of normal distribution and can be difficult to identify with a t-test. Instead, we used F-test of variance to identify high-variance genes and the non-parametric two-sample Kolmogorov-Smirnov Test to test differences in both location and shape of the distributions.

### 4.4. Identification of Test Genesets

In this study, we investigated the hypothesis that genes critical to the development of the liver are poorly regulated or preferentially activated in HCV-cirrhosis and HCV-HCC. There are five main stages of liver development: hepatic fate specification, hepatoblast migration, liver bud growth, hepatocyte/biliary differentiation, and maturation. We identified 179 genes (via literature review) that are experimentally determined to be critical regulators necessary for normal liver development (See Supplementary Table S2 for a complete list). We also wished to determine whether liver development genes are more likely than their non-liver paralogs (which have highly similar functions in the development of other tissue types) to be dysregulated in liver tumors. We identified 26 such genes that are not normally expressed in either embryonic or adult liver tissue (Supplementary Table S1).

## 5. Conclusions

In summary, there is growing awareness that hepatocarcinogenesis shares many features with liver organogenesis. Recent reviews have compared the pathways that appear to be involved in both development and cancer [66,67]. We show here, for the first time to our knowledge, that tumors dysregulate the genes specific to their own development rather than closely related genes in the same pathways. We further demonstrate that these closely related paralogs, which have established roles in cancers of other tissues, remain under good transcriptional control in HCV-induced cirrhosis and HCC even in late-stage tumors. We have found that the earliest signals responsible for guiding the initiation and development of the embryonic liver are correlated with the development of cirrhosis and HCC due to chronic Hepatitis C infection, but do not appear to be involved in the progression from early to late stage cancer. These observed patterns of expression were validated in an independently collected HCV-HCC microarray experiment. By applying what is known about the control of these genes in normal liver development and their aberrant control in HCC, we can begin to model knock out and deletion experiments to further define how these pathways can be interrupted or stimulated to impact HCC occurrence or recurrence.

## Supplementary Material

**Table S1:** Liver development genes compared to their non-liver paralogs. A: one-sided K-S test of identical distribution comparing HCC to normal samples; B: on-sided K-S test in the Wurmbach dataset; C: K-S test comparing the liver development gene to it’s non-liver paralog.

Liver development gene	Expression in normal adult liver	Non-liver paralog	Non-liver gene, tumor *vs*. normal (p-value)	Non-liver gene, tumor *vs*. normal (p-value)	Liver *vs*. non-liver gene in tumors (p-value)
			VCU data	Wurmbach data	VCU data
			A	B	C
ACVR2A	+	AMHR2	0.18	0.009	0.14
BMP2	+	BMP3	0.014	0.57	9.9 × 10^−10^
BMP4	+	BMP3	0.014	0.57	0.0091
CDH1	++	CDH3	0.20	0.007	1.7 × 10^−6^
ELF5	-	SPDEF	0.003	0.29	5.2 × 10^−8^
FGF1	-	FGF3	0.73	0.37	0.0023
FGF2	+	FGF3	0.73	0.37	1.7 × 10^−6^
FGF7	+	FGF12	0.81	0.20	1.7 × 10^−6^
FGF8	-	FGF17	0.90	0.69	0.14
FOXA1	++	FOXB1	0.02	0.97	2.2 × 10^−16^
FOXA2	++	FOXD2	0.06	0.72	1.2 × 10^−12^
GATA4	++	GATA1	0.05	0.18	2.4 × 10^−10^
GATA6	+	GATA1	0.05	0.18	1.7 × 10^−6^
GPC3	-	GPC4	0.29	0.22	1.1 × 10^−11^
HHEX	++	VENTX	0.002	0.32	3.6 × 10^−5^
HLX	+	BARX1	0.02	0.02	0.00049
IL6ST	+++	IL12RB2	0.25	0.59	7.3 × 10^−6^
KIT	+	FLT3	0.87	0.09	0.0011
KRT19	+	KRT17	0.12	0.59	3.7 × 10^−8^
LHX2	+	LHX1	0.31	0.55	2.7 × 10^−6^
MET	++	MST1R	0.19	0.006	0.00026
MMP7	+	MMP10	0.59	0.07	2.2 × 10^−16^
MMP12	-	MMP10	0.59	0.07	0.0012
MMP14	+	MMP10	0.59	0.07	1.2 × 10^−7^
MMP19	+	MMP10	0.59	0.07	0.0025
MMP2	+	MMP10	0.59	0.07	2.2 × 10^−16^
NR5A2	++	NR5A1	0.32	0.37	2.2 × 10^−16^
NRTN	+	PSPN	064	0.36	2.2 × 10^−16^
RXRA	+++	RXRG	0.05	0.15	2.2 × 10^−16^
SOX9	*	SOX1	0.27	0.05	4.4 × 10^−16^
SOX17	+	SOX11	0.01	0.26	0.0005
TBX3	++	TBX2	0.34	0.02	5.6 × 10^−7^
WT1	-	EGR4	0.14	0.009	3.6 × 10^−5^

**Table S2:** All liver development genes tested and their relative expression in HCV-cirrhosis, HCC-cirrhotic tissues, early stage HCC and late stage HCC. Fold-change (FC) is relative to normal controls.

Probe Set ID	Gene name	Mean FC HCV-CIR	Mean FC Early HCC	Mean FC late HCC
NM_001105_at	ACVR1	1.01	1.09	0.90
NM_004302_at	ACVR1B	0.92	0.86	0.87
NM_001616_at	ACVR2A	0.82	0.96	0.96
NM_001106_at	ACVR2B	0.50	0.58	0.59
NM_001663_at	ARF6	1.18	1.11	1.11
NM_032199_at	ARID5B	3.62	2.13	1.86
NM_001880_at	ATF2	0.93	1.27	1.41
NM_006856_at	ATF7	0.92	0.93	0.96
NM_006395_at	ATG7	0.88	0.91	0.97
NM_001200_at	BMP2	1.56	1.42	1.32
NM_001202_at	BMP4	0.77	1.02	1.09
NM_004329_at	BMPR1A	1.38	1.37	1.32
NM_001203_at	BMPR1B	0.97	1.01	1.05
NM_001204_at	BMPR2	1.00	1.09	1.02
NM_001728_at	BSG	1.28	1.23	1.31
NM_014333_at	CADM1	1.60	1.68	1.89
NM_053056_at	CCND1	1.38	1.34	0.81
NM_001759_at	CCND2	1.16	1.16	1.22
NM_057749_at	CCNE2	1.17	1.81	2.16
NM_004360_at	CDH1	1.74	1.49	1.41
NM_004364_at	CEBPA	0.61	0.93	0.95
NM_005454_at	CER1	0.81	0.89	0.99
NM_006079_at	CITED2	2.74	1.93	1.63
NM_001845_at	COL4A1	5.29	4.22	4.07
NM_001846_at	COL4A2	3.84	2.59	2.30
NM_000091_at	COL4A3	1.10	1.04	1.01
NM_000092_at	COL4A4	1.54	1.16	1.24
NM_000495_at	COL4A5	1.38	1.79	1.42
NM_001847_at	COL4A6	0.93	0.95	0.95
NM_000096_at	CP	0.90	0.87	0.66
NM_001893_at	CSNK1D	1.47	1.05	1.09
NM_001904_at	CTNNB1	0.86	1.18	1.05
NM_012242_at	DKK1	1.07	3.86	1.75
NM_001422_at	ELF5	0.89	0.97	1.01
NM_002354_at	EPCAM	14.99	14.35	5.72
NM_004448_at	ERBB2	1.37	1.28	1.37
NM_000800_at	FGF1	1.10	1.02	1.07
NM_002006_at	FGF2	0.98	0.99	0.93
NM_002009_at	FGF7	1.54	1.28	1.17
NM_006119_at	FGF8	0.99	1.09	1.09
NM_015850_at	FGFR1	1.11	1.04	0.98
NM_000141_at	FGFR2	4.22	2.38	1.68
NM_002026_at	FN1	1.08	1.17	1.28
NM_004496_at	FOXA1	0.41	0.53	0.42
NM_021784_at	FOXA2	0.61	0.67	0.66
NM_202002_at	FOXM1	0.87	1.04	1.36
NM_006350_at	FST	1.32	2.08	1.95
NM_005860_at	FSTL3	3.39	1.64	1.57
NM_000151_at	G6PC	1.53	1.30	1.13
NM_002052_at	GATA4	0.67	0.75	0.81
NM_005257_at	GATA6	2.84	1.67	0.91
NM_001495_at	GFRA2	1.02	1.01	1.01
NM_004484_at	GPC3	2.07	7.77	10.13
NM_002086_at	GRB2	0.86	0.94	0.96
NM_013372_at	GREM1	1.25	3.66	2.09
NM_021973_at	HAND2	2.23	1.71	1.35
NM_004494_at	HDGF	1.06	1.16	1.25
NM_014571_at	HEYL	1.09	1.16	1.13
NM_000601_at	HGF	1.18	1.01	0.98
NM_002729_at	HHEX	0.83	1.27	1.10
NM_021958_at	HLX	1.16	1.14	1.06
NM_003483_at	HMGA2	0.93	0.94	1.12
NM_002129_at	HMGB2	2.53	3.26	3.46
NM_000545_at	HNF1A	0.72	0.87	0.86
NM_000458_at	HNF1B	1.33	0.99	1.11
NM_000457_at	HNF4A	0.86	0.96	1.04
NM_006896_at	HOXA7	0.95	0.98	0.97
NM_005529_at	HSPG2	1.22	1.05	1.04
NM_012405_at	ICMT	0.84	0.82	0.97
NM_002166_at	ID2	0.83	0.77	0.68
NM_002167_at	ID3	2.36	1.66	1.28
NM_000612_at	IGF2	1.03	0.98	0.44
NM_002184_at	IL6ST	1.05	0.96	0.93
NM_002191_at	INHA	1.07	1.08	1.05
NM_002192_at	INHBA	0.86	1.32	1.26
NM_002193_at	INHBB	3.14	1.72	1.48
NM_005538_at	INHBC	0.66	0.77	0.77
NM_031479_at	INHBE	0.70	0.67	0.39
NM_005544_at	IRS1	0.88	0.99	1.01
NM_003749_at	IRS2	1.40	1.09	1.09
NM_002204_at	ITGA3	1.38	1.19	1.34
NM_002205_at	ITGA5	1.13	0.94	0.95
NM_000210_at	ITGA6	1.44	1.83	1.76
NM_033668_at	ITGB1	0.83	0.91	0.92
NM_000213_at	ITGB4	1.04	1.06	1.09
NM_000214_at	JAG1	2.22	2.06	1.91
NM_000222_at	KIT	2.05	1.61	1.49
NM_001300_at	KLF6	3.97	2.66	1.73
NM_004985_at	KRAS	0.92	0.80	0.71
NM_002276_at	KRT19	5.71	2.96	1.86
NM_000426_at	LAMA2	4.34	2.31	1.76
NM_000227_at	LAMA3	1.36	1.43	1.81
NM_002290_at	LAMA4	1.12	1.27	1.26
NM_005560_at	LAMA5	1.07	0.98	1.02
NM_002291_at	LAMB1	3.44	2.45	1.91
NM_002292_at	LAMB2	1.14	0.93	0.82
NM_000228_at	LAMB3	0.86	0.93	0.92
NM_007356_at	LAMB4	0.95	1.06	1.05
NM_002293_at	LAMC1	1.43	1.58	1.66
NM_005562_at	LAMC2	1.07	0.96	1.01
NM_006059_at	LAMC3	2.33	1.41	1.30
NM_016269_at	LEF1	1.19	1.16	1.20
NM_004789_at	LHX2	1.11	0.87	0.77
NM_003010_at	MAP2K4	1.07	0.97	0.95
NM_004834_at	MAP4K4	0.92	1.29	1.33
NM_001315_at	MAPK14	0.87	1.02	1.06
NM_002750_at	MAPK8	0.98	1.00	1.09
NM_002391_at	MDK	1.71	2.69	2.42
NM_000245_at	MET	0.79	1.12	1.29
NM_002421_at	MMP1	1.31	1.40	3.07
NM_005940_at	MMP11	1.03	1.07	1.09
NM_002426_at	MMP12	1.11	9.73	21.08
NM_004995_at	MMP14	0.85	0.85	0.92
NM_002428_at	MMP15	0.73	0.71	0.73
NM_005941_at	MMP16	0.92	0.99	1.01
NM_016155_at	MMP17	0.66	0.84	0.85
NM_002429_at	MMP19	1.48	1.18	1.21
NM_004530_at	MMP2	5.14	4.57	3.03
NM_022468_at	MMP25	0.92	0.84	0.93
NM_002423_at	MMP7	7.08	5.04	5.29
NM_004994_at	MMP9	1.22	2.45	4.35
NM_020998_at	MST1	0.93	0.62	0.53
NM_005955_at	MTF1	0.89	0.95	0.98
NM_005378_at	MYCN	0.95	1.08	1.07
NM_002487_at	NDN	2.15	1.28	1.25
NM_000267_at	NF1	0.91	0.89	0.92
NM_003998_at	NFKB1	1.28	1.07	1.10
NM_002508_at	NID1	1.11	0.90	0.86
NM_014360_at	NKX2-8	0.99	1.15	1.06
NM_024408_at	NOTCH2	1.05	0.82	0.86
NM_000435_at	NOTCH3	1.08	1.06	1.12
NM_003822_at	NR5A2	0.57	0.92	0.75
NM_005011_at	NRF1	1.11	1.09	1.08
NM_004558_at	NRTN	0.33	0.39	0.36
NM_004498_at	ONECUT1	1.03	1.01	0.92
NM_004852_at	ONECUT2	0.88	0.98	0.93
NM_020530_at	OSM	1.16	1.17	1.18
NM_006191_at	PA2G4	1.06	0.93	1.11
NM_005392_at	PHF2	1.09	0.90	0.87
NM_006218_at	PIK3CA	1.11	1.11	1.04
NM_181504_at	PIK3R1	1.22	0.87	0.77
NM_002763_at	PROX1	0.76	0.67	0.43
NM_002825_at	PTN	2.08	1.56	1.37
NM_002957_at	RXRA	0.52	0.52	0.53
NM_012432_at	SETDB1	0.98	1.11	1.16
NM_003015_at	SFRP5	2.50	1.71	1.41
NM_005901_at	SMAD2	1.36	1.44	1.48
NM_005902_at	SMAD3	0.90	0.97	1.00
NM_005359_at	SMAD4	0.97	0.99	0.98
NM_005903_at	SMAD5	0.87	0.93	0.90
NM_005585_at	SMAD6	1.17	1.06	0.99
NM_005904_at	SMAD7	2.94	1.77	1.25
NM_005905_at	SMAD9	0.97	0.98	0.97
NM_022454_at	SOX17	1.20	1.08	0.96
NM_000346_at	SOX9	4.81	2.99	3.12
NM_000582_at	SPP1	9.75	9.54	16.41
NM_003137_at	SRPK1	0.76	0.92	1.05
NM_003150_at	STAT3	0.83	0.57	0.50
NM_018234_at	STEAP3	0.57	0.57	0.34
NM_016569_at	TBX3	0.78	1.55	1.76
NM_003200_at	TCF3	1.03	1.07	1.10
NM_000660_at	TGFB1	3.13	1.90	1.75
NM_003238_at	TGFB2	0.95	0.99	0.98
NM_003239_at	TGFB3	1.00	0.93	0.97
NM_004612_at	TGFBR1	0.91	1.03	1.08
NM_003242_at	TGFBR2	1.51	1.39	1.17
NM_003243_at	TGFBR3	1.19	0.67	0.56
NM_003255_at	TIMP2	1.43	1.50	1.33
NM_003256_at	TIMP4	0.92	0.88	0.89
NM_000594_at	TNF	0.90	0.91	0.98
NM_015542_at	UPF2	1.02	1.23	1.26
NM_003380_at	VIM	5.53	4.74	5.16
NM_000378_at	WT1	1.08	1.09	1.08
NM_005080_at	XBP1	0.64	0.86	0.73
NM_006106_at	YAP1	1.06	0.96	0.75
NM_015642_at	ZBTB20	2.03	1.38	1.25
NM_014943_at	ZHX2	1.11	1.02	1.09
NM_004773_at	ZNHIT3	1.25	1.40	1.38

**Table S3:** Individual fold-change for BMP inhibitors in HCC samples.

Sample	FSTL3	GPC3	GREM1	FST
T1_377	1.7	8.9	1.5	2.0
T1_527	2.0	2.2	1.1	1.1
T1_607	2.6	1.6	1.1	0.8
T2_116	1.7	17.2	0.9	4.3
T2_184	1.3	1.7	13.7	1.0
T2_309	1.6	2.5	1.0	1.7
T2_334	2.1	3.6	1.0	1.9
T2_342	0.9	15.4	4.6	3.9
T2_388	3.2	1.9	27.5	0.9
T2_422	1.0	1.7	1.4	2.8
T2_451	2.5	1.8	0.9	1.6
T2_507	1.7	1.3	1.1	1.1
T2_524	2.1	2.7	1.2	1.3
T2_550	1.2	23.5	1.4	3.8
T2_588	1.1	27.7	2.4	1.2
T2_614	1.1	1.1	1.2	2.1
T2_657	1.1	6.3	12.7	1.7
T2_666	1.5	12.4	1.2	1.8
T2_718	1.1	1.0	0.9	1.8
T2_728	1.1	10.9	13.0	1.1
T2_753	3.0	1.5	2.6	0.5
T2_753B	1.8	0.8	1.1	0.3
T2_787	1.2	2.5	1.4	1.7
T2_R2926	1.4	41.5	8.0	6.3
T2_R2927	1.4	1.4	0.9	3.8
T2_R2928	1.3	1.3	1.1	2.9
T2_R2929	1.0	14.3	0.9	2.2
T2_ R3502	2.0	1.9	1.1	0.8
T2_VM3	2.3	3.0	0.9	0.7
T2_VM4	2.7	1.1	1.5	0.7
T3_256	3.2	1.4	0.9	1.4
T3_297	1.5	2.5	1.0	4.4
T3_329	1.2	14.2	1.1	3.1
T3_358	1.8	1.0	1.0	1.4
T3_411	1.1	1.4	6.3	0.9
T3_584	1.4	15.2	1.0	4.2
T3_627	1.1	50.3	1.3	1.8
T4_300	2.1	12.0	1.6	1.9
T4_393	0.9	5.9	3.0	3.3
T4_400	1.2	2.3	1.3	0.7
T4_531	1.2	1.1	1.1	2.6
T4_552	1.0	11.1	0.8	0.8
T4_810	1.5	25.1	1.0	1.8
T4_R2858	1.3	21.3	1.1	4.4
T4A_VM1	2.3	3.0	1.0	1.1
T4B_324	1.0	24.5	0.9	1.3
T4B_353	1.0	1.2	11.1	1.7
T4B_381	1.9	1.6	1.3	1.3
T4B_382	1.5	1.9	1.4	2.2
